# Utilising telehealth to support exercise and physical activity in people with Parkinson disease: a program evaluation using mixed methods

**DOI:** 10.1186/s12913-023-09194-0

**Published:** 2023-03-07

**Authors:** Allyson Flynn, Elisabeth Preston, Sarah Dennis, Colleen G. Canning, Natalie E. Allen

**Affiliations:** 1 Sydney School of Health Sciences, Faculty of Medicine and Health, The University of Sydney, Sydney, Australia; 2 Discipline of Physiotherapy, Faculty of Health, University of Canberra, Canberra, Australia; 3 South Western Sydney Local Health District, Liverpool, Australia; 4 Ingham Institute of Applied Medical Research, Liverpool, Australia

**Keywords:** Telemedicine, Parkinson disease, Exercise therapy, Physiotherapy

## Abstract

**Background:**

Physical activity and exercise play a key role in managing Parkinson disease. This study aimed to: 1) determine if physiotherapy supported by telehealth helped people with Parkinson disease (PwP) to adhere to a home-based exercise program and maintain their physical activity; and 2) understand their experiences of using telehealth during the COVID-19 pandemic.

**Methods:**

A mixed methods program evaluation involving a retrospective file audit from a student-run physiotherapy clinic and semi-structured interviews exploring participants’ experiences of telehealth. Ninety-six people with mild to moderate disease received home-based telehealth physiotherapy for 21 weeks. The primary outcome was adherence to the prescribed exercise program. Secondary outcomes were measures of physical activity. Interviews were conducted with 13 clients and seven students and analysed thematically.

**Results:**

Adherence to the prescribed exercise program was high. The mean (SD) proportion of prescribed sessions completed was 108% (46%). On average clients spent 29 (12) minutes per session, and 101 (55) minutes per week exercising. Physical activity levels were maintained, with clients taking 11,226 (4,832) steps per day on entry to telehealth, and 11,305 (4,390) steps per day on exit from telehealth. The semi-structured interviews identified important features of a telehealth service required to support exercise; a flexible approach of clients and therapists, empowerment, feedback, a therapeutic relationship, and mode of delivery.

**Conclusions:**

PwP were able to continue exercising at home and maintain their physical activity when physiotherapy was provided via telehealth. The flexible approach of both the client and the service was imperative.

**Supplementary Information:**

The online version contains supplementary material available at 10.1186/s12913-023-09194-0.

## Background

Physical activity and exercise have a key role in the management of Parkinson disease. Physical activity comprises any body movement produced by skeletal muscle and includes leisure activities and housework [1]. Exercise is a subset of physical activity which is planned, structured and purposeful with the intention of improving/maintaining physical fitness [1]. Previous research suggests that home-based exercise is effective in improving motor impairments [2], balance related activities and walking speed in people with mild to moderate disease [3]. A vital feature of effective home-based exercise is the ability to monitor and progress the exercise.

Telehealth, where the provision of health care is via telephone, and/or video calls and/or smartphone apps, is increasingly being used to monitor home-based exercise [4]. During the COVID-19 pandemic there has been a rapid uptake of telehealth into clinical practice [5]. The advantages of telehealth include: reduced travel time for PwP, increased accessibility especially for people in rural settings where specialised services are limited, and reduced cost for both PwP and therapists [4, 5]. Video calls also enable the therapist to view the home environment and assist PwP to set up exercises safely. Recent small studies have shown that individual video calls are a feasible and safe way to provide telehealth to people with mild to moderate Parkinson disease [6, 7]. However, while potentially effective, there are concerns that telehealth results in reduced personal contact, and that a lack of accessibility to appropriate technology, or skill required to use the technology, may limit its impact [4, 5]. Furthermore, little is known about therapists and PwP experiences of using telehealth to deliver and complete exercise programs.

During the COVID-19 pandemic PwP experienced a decrease in physical activity and overall health and wellbeing [8–10]. For many PwP, home-based physical activity and exercise has been the only option available as access to in-person physiotherapy, fitness centres and exercises classes was limited. This provided a unique opportunity to further explore the capabilities of multiple modes of telehealth (telephone calls, individual video calls and group video calls) and gain an understanding of the experiences of PwP and therapist when using telehealth.

Given the potential for telehealth to provide accessible, sustainable and efficient physiotherapy into the future, this study aimed to:

- Describe how physiotherapy supported by different modes of telehealth helped PwP to adhere to home-based exercise and maintain physical activity during a Covid-19 lockdown, while unable to attend in-person services.
- Understand the experiences of PwP and physiotherapy students when using telehealth

## Method

### Study design

A mixed methods program evaluation examined the findings from a retrospective file audit and semi-structured interviews, using a concurrent complementary approach. This involved simultaneously conducting: 1) a retrospective file audit of clinical records from a university student-run physiotherapy clinic for people with PwP in a metropolitan region of Australia; 2) semi-structured interviews to explore the experiences of the PwP who had undertaken telehealth; 3) semi-structured interviews to explore the experiences of the students who delivered telehealth. The interviews were designed to clarify and enhance the file audit results as well as guide future clinical practice.

### Description of telehealth service

Community dwelling people diagnosed with Parkinson disease who were attending or referred to a weekly in-person group exercise class at the university clinic were rapidly transitioned to home-based telehealth physiotherapy. Telehealth was provided for 21 weeks between March and August 2020 when in-person services were restricted.

All clients were prescribed an individualised exercise program targeting balance and mobility. For existing clients, students were supervised by the physiotherapist (AF) to select exercises from the PhysioTherapy eXercises website [11] which were similar to those the clients had been completing in the clinic (see example in the Supplementary material). These exercises were taught to the client over the telephone. New clients received up to three individual video calls using Coviu (Coviu Global Pty Ltd, Australia) before transitioning to telephone calls. The video calls were used to assess the client in their home environment (one call) and to teach the home exercise program (up to two calls). When selecting exercises, consideration was given to each client’s goals, ability, and available equipment. Exercise programs were designed to take 30 to 50 min, three times a week. All clients were provided with written information about how to exercise safely at home and were asked to record daily how many minutes they spent completing the exercise program on a recording sheet.

Clients were supported to continue the exercise program via weekly telephone calls, which lasted for approximately 10–15 min each. The client reported if they had been completing the exercise program and any challenges or barriers they had experienced. If barriers were identified, the client and therapist developed solutions to overcome these. If required, a video call was conducted to assist with rectifying any problems. Clients were also provided with feedback and encouragement on the amount of exercise completed and the exercise program was monitored and progressed by asking the client how challenging each exercise was to complete. Following discussions with the physiotherapist, student and client, if indicated, exercises were progressed by increasing the dose (e.g., increasing the number of repetitions from 10 to 15 or increasing the time performing the exercise), or making the exercise more challenging (e.g., by adding weight, reducing the base of support or changing the exercise). Every three to five weeks clients were sent an updated exercise program which reflected any changes made during the weekly telephone calls. Where possible telephone calls were conducted at a similar time each week which corresponded to when the client would have been attending the clinic in-person, thereby providing clients with an approximate time to expect the call.

Group video calls where three to four clients completed exercises at the same time under the instruction of the therapist/students were offered weekly to some clients. Selection for group video calls was based on client safety and willingness to be involved. The physiotherapist demonstrated each exercise while all the clients watched their screen. All the clients then completed the exercise at the same time with the physiotherapist and student watching the screen and providing feedback as the client was completing the exercise. Further detail about the telehealth service, including safe delivery of video calls, is in the supplementary material.

Objective and self-reported measures of physical activity were collected within the first 5-weeks of the telehealth service and again 1–3 weeks prior to the client returning to the in-person classes, where possible. Physical activity was measured objectively using the ActiGraph triaxial accelerometer (ActiGraph GT3X + , ActiGraph, Penascola). Clients wore the accelerometer on their right hip during waking hours for five consecutive days. Data from clients with at least three valid days, including nine or more hours of daily wear time were included. The ActiLife 6 software was used to analyse the data. The Freedson adult algorithm was used to calculate the steps taken per day and percentage of time sedentary or completing light, moderate and vigorous physical activity per day [12]; and the percentage of time in walking speeds of less than 1.04 m/s, between 1.05 m/s to 1.30 m/s and above 1.31 m/s was calculated using the Nero algorithm [13]. Self-reported physical activity was collected via the International Physical Activity Questionnaire – Elderly (IPAQ – E) and reported as metabolic equivalent/minutes/week [14].

### Retrospective audit

A retrospective file audit was conducted of all clients who received telehealth between March and August 2020. Clients who received less than four telehealth phone calls were excluded from the audit.

A data collection tool was specifically designed to collect the following information: number and type of telehealth sessions completed, client reported adherence to the home-based exercise program, reported as number of sessions and time spent completing the program per week. Where available measures of physical activity were extracted. Background information (age, year of diagnosis, fall risk score [15], freezing of gait (New Freezing of Gait Questionnaire) [16], and disease severity were also extracted.

The primary outcome was adherence to the exercise program, quantified as the percentage of prescribed exercise sessions completed and the time spent completing the program per week. Adherence and client characteristics were analysed using descriptive statistics. The difference between pre and post physical activity was examined using paired t-tests. SPSS Statistics (version 25, IBM Corp, Armonk, NY, USA) was used for all quantitative analyses.

### Semi structure interviews

Purposive sampling was used to recruit participants to ensure information was gathered on all aspects of the telehealth service. Therefore, a selection including: physiotherapy students who had provided all three modes of telehealth, existing clients who received telephone calls, individual or group video calls, new clients to the clinic (not attended in-person prior to telehealth), and clients who declined to participate in telehealth were invited to participate.

Semi-structured interviews were used to collect data regarding experience of telehealth. Interview guides were developed for both the clients and student interviews (see supplementary material Box 1). The interviewer was an experienced qualitative researcher and physiotherapist, who had previously worked in the clinic, but was not involved in providing telehealth. Interviews were audio recorded and transcribed verbatim, and field notes including initial observations from the interview were made.

Transcribed interviews were analysed inductively using a thematic framework method [17], and Nvivo v 12 software. Two researchers (AF, SD) immersed themselves in the data by independently reading the transcripts, listening to the interviews and discussing with the interviewer. Researchers then agreed on initial codes. Transcripts were coded inductively by one researcher (AF), with further discussion between the researchers to refine codes. Coded data were interpreted, and key themes identified (AF, SD). Themes were then reviewed and refined by all researchers and presented to participants for member-checking. Feedback from participants supported the key themes and findings identified. Creditability, dependability and transferability were achieved through logical and traceable analysis involving discussions between authors, member checking of the analysis and clearly outlining the setting [18]. To ensure confirmability quotes from the clients and students have been reported in the results to demonstrate how the researchers’ interpretations and findings were derived from the dataset [18].

## Results

**Fig. 1:**
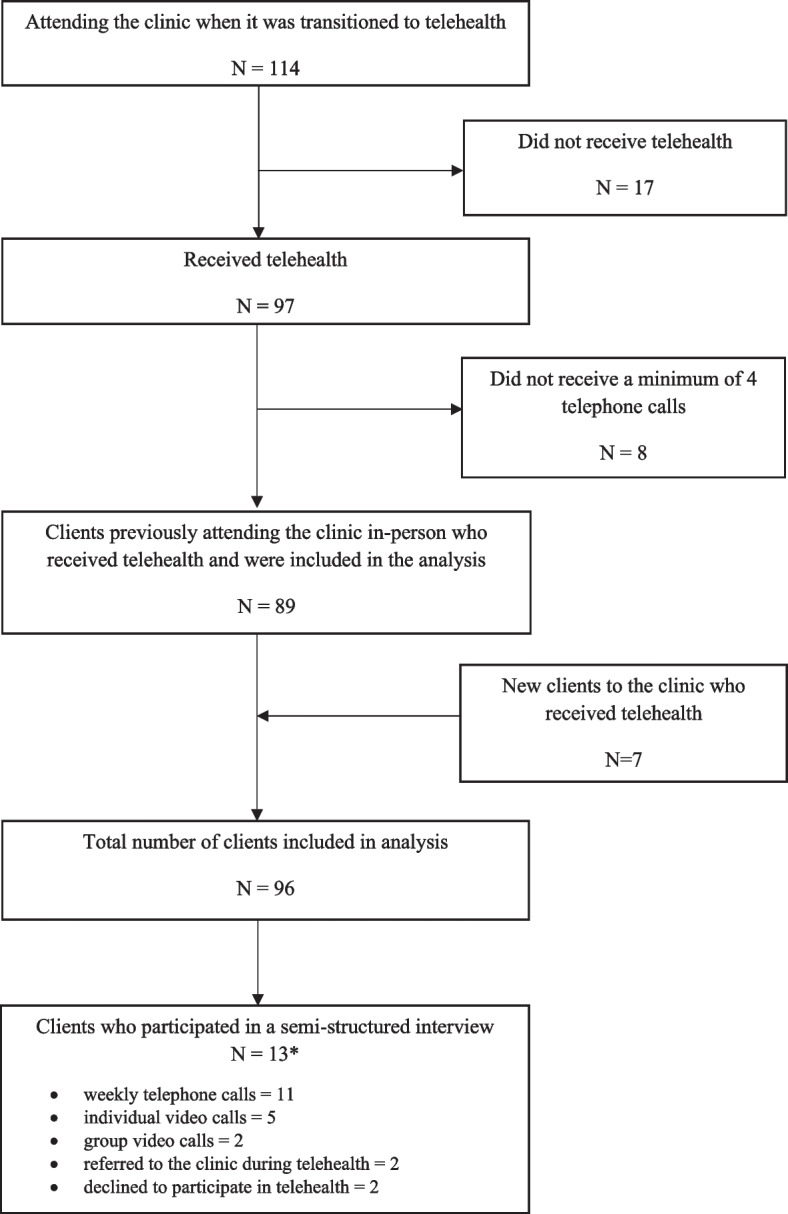
Flow of clients *Clients may have participated in more than one mode of telehealth

Data were available for 89 (78%) clients of the 114 who attended the clinic prior to telehealth. A further seven people were referred to the clinic during the telehealth service (Fig. 1) so data were available for a total of 96 clients. All clients had mild to moderate Parkinson disease (Hoehn and Yahr I-III), 29% reported freezing of gait and 33% had a high fall risk (Table 1).

**Table 1:** Client characteristics at baseline. Mean (SD) or number (%)

	*n=*96
Age (years)	74 (7)
Gender (female)	35 (36)
Disease duration (years)^a^	6 (4)
Hoehn and Yahr [19]	
Stage 1, n (%)	38 (40)
Stage 2, n (%)	31 (32)
Stage 3, n 9%)	27 (28)
Falls risk^b^	
Low, n (%)	22 (24)
Moderate, n (%)	37 (40)
High, n (%)	33 (36)
Freezing of gait n (%)^c^	27 (29)
NFOGQ^d^	4 (6.6)
Time attending clinic	
Longer 1 year, n (%)	65 (68)
Between 2 -12 months, n (%)	17 (18)
Started during telehealth, n (%)	7 (7)

^a^*n =* 84 (9 people unsure of year of diagnosis and unable to calculate years for 3 people as diagnosed in 2020 and no month recorded)

^b^*n =* 92, Falls risk calculated using three step clinical prediction tool (falling in the last year, self-reported gait speed, freezing of gait in the last month) [15]

^c^*n =* 94 not recorded for 2 clients

^d^*n =* 62

*NFOGQ*  New freezing of gait questionnaire

There were 28 individual video calls for 17 (18%) clients conducted during the 21 weeks of telehealth. Group video calls were conducted with 12 (13%) clients. There were two adverse events; both were non-injurious falls while exercising. One occurred during a group video call.

### Adherence and physical activity

Clients were provided on average (SD) 10 (3) weeks of telehealth, with a staggered return to in-person services at the end of lockdown. Overall adherence to the exercise program was high, with 108% (46) of prescribed sessions completed, indicating that some clients did more than the prescribed three sessions per week. The average length of time per exercise session was 29 (12) minutes, range 9 – 82 min, and time completing exercise per week was 101 (55) minutes, range 27 – 290 min (Table 2) indicating clients spent slightly less time exercising per session than prescribed.

**Table 2:** Number of weeks of telehealth with data and number of sessions completed

	**Mean (SD)** **or** **number (%)**	**Range**
Number of weeks of telehealth	10 (3)	4—17
Total number of completed sessions	35 (19)	5—96
Percentage of completed sessions	108 (46)	29—231
Number of clients who completed		
< 50% of sessions	8 (8)	
50% to 100% sessions	39 (41)	
101% to 150% sessions	32 (33)	
> 150% of sessions	17 (18)	

Number of clients = 96, % = percentage

Overall, clients maintained their pre-existing levels of physical activity, with clients taking 11,226 (4,832) steps per day on entry to telehealth, and 11,305 (4,390) steps per day on exit from telehealth. There was a very small reduction in time spent in moderate and vigorous physical activity (mean difference -0.5%, 95% CI -1.0 to 0.0) at the end of telehealth. There was no difference in any of the other physical activity outcomes (Table 3).

**Table 3:** Physical activity, mobility and balance and psychological wellbeing measures for clients pre and post intervention. Mean (SD), 95% CI

	**Number**	**Pre**	**Post**	**Difference**	
	**Clients**	**Mean (SD)**	**Mean (SD)**	**Mean (SD)**	**95% CI**
**Physical Activity**					
Average number of steps per day	42	11 226 (4832)	11 305 (4390)	79 (1903)	-514 to 672
% time sedentary	42	69.4 (13.0)	68.9 (11.7)	-0.5 (5.8)	-2.3 to 1.3
% time light PA	42	27.6 (11.7)	28.7 (11.1)	1.0 (5.4)	-0.7 to 2.7
% time MVPA	42	3.0 (3.6)	2.5 (3.1)	-0.5 (1.6)	-1.0 to 0.0
% time walking speed < 1.04 m/s	42	94.4 (4.5)	94.8 (4.2)	0.4 (2.0)	-0.3 to 1.0
% time walking speed 1.05 m/s to 1.30 m/s	42	3.9 (2.7)	3.8 (2.7)	-0.1 (1.6)	-0.6 to 0.4
% time walking speed > 1.31 m/s	42	1.7 (2.3)	1.4 (1.9)	-0.3 (1.4)	-0.7 to 0.2
IPAQ-E (METs/min/week)^a^	27	3557 (3205)	3774 (3232)	218 (3386)	-1121 to 1557

*IPAQ-E*  International Physical Activity Questionnaire – Elderly, *METs*  Metabolic equivalent, *%*  Percentage, *MVPA*  Moderate and vigorous physical activity, *PA*  Physical activity

^a^Higher score is better

### Experiences of telehealth

All participants who were invited to be interviewed accepted. Interviews were conducted with 13 clients and seven students (from a possible 22 students who provided telehealth). Of the 13 clients who participated 11 received weekly telephone calls, five individual video calls, two group video calls, two were new to the clinic and two declined telehealth. All students provided telephone calls, individual and group video calls.

**Fig. 2:**
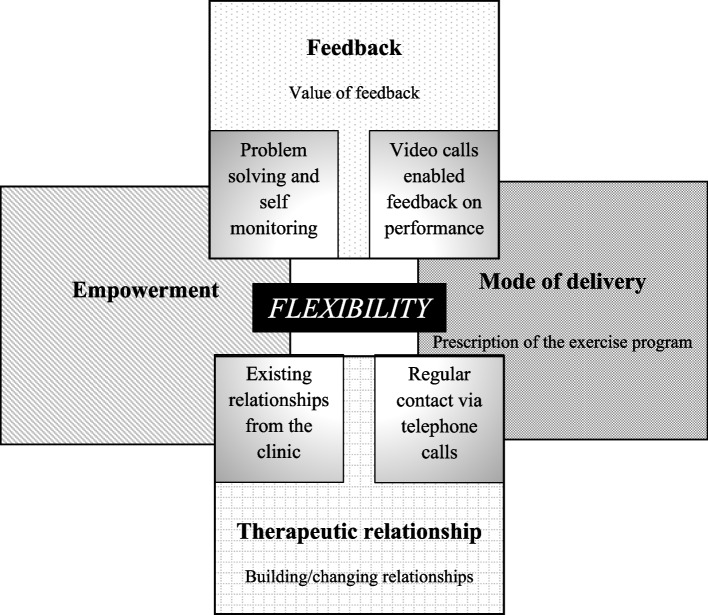
Illustration of the interaction of the themes

The qualitative analysis provided important insights into the features of the telehealth that facilitated clients to maintain their exercise and physical activity. We identified one overarching theme of flexibility and four themes of empowerment, value of feedback, mode of delivery of telehealth and the therapeutic relationship (Table 4). The interaction between these themes and their subthemes is seen in Fig. 2. The clients’ experience of telehealth was influenced by the flexible approach of both themselves and the service. Without the flexibility shown by the clients (to embrace and engage in the telehealth) and physiotherapist and students (to embrace and deliver telehealth in multiple ways) the telehealth service would not have occurred. Clients who were flexible in their approach adapted quickly to exercising at home, completing the program in a way convenient to them. They identified an appropriate space to exercise in, incorporated exercise into their daily routine and engaged regularly with telehealth. From a service perspective, the more flexible the type of telehealth (i.e., telephone, video) offered the more it met the needs of the client and enhanced their experience.

**Table 4:** Summary of themes, subthemes and supporting quotes

Theme	Subtheme	Supporting quotes
Empowerment	Problem solving ways to complete the exercise	“I did on a position on the steps to the back deck, where I was looking straight at a glass door, so it was the same effect as having a mirror. I could see how high I was lifting my knee and all of that sort of thing.” (Client (C) C7) “What I did was I just kind of incorporated them into my day” (C9) “Well I’ve set up obstacles out on the veranda, so I’ve got a reasonably long veranda going onto a footpath …… And then I set up the doggy trampoline at the back wall and used the ball to throw against the wall.” (C1) “But you just improvise, and the other thing is just to vary it a bit, sometimes you do it a little bit differently. But still trying to concentrate on the core part of the exercise, which in that case might be balance or stretching out or something like that.” (C13)
	Developing self-monitoring strategies	“I really like to have a fairly detailed recording sheet, so that I can compare my progress.” (C5) “My friend has actually given me her Fitbit. So that’s quite good too, because you think if I get on the treadmill, I can actually see how many steps I’m doing,…….. So that’s been motivating, to just help with doing that exercise.” (C5)
Value of feedback	Feedback provided via telephone calls was limited	“I think I’m doing it right; I’m never entirely 100 percent sure that I am, of course, because there’s no one there telling me that that’s right or not right.” (C3) “….then you saw them face-to-face and thought, you’re doing something completely different to what I thought you were doing.” (Student (S)7) “I guess that’s the other thing that’s hard over telehealth is like, like self-reported exercise, it’s really difficult to know what they’re actually doing and whether they’re doing it correctly even though they think they are.” (S6)
	Video calls enabled feedback on performance of the exercises	“…kind of the video calls, when you see people doing it you realise how much more feedback you can give when you actually see it.” (S4) “think the video calls were really great because, and we got to see what people were actually doing and give them some specific feedback, which was really helpful for them.” (S7) “But then when we went to video later on, it was so much easier because you could demonstrate with the video, what you’re talking about. That was… so I found that the video was terrific.” (C8) “In a sense it didn’t feel any different from being assessed at the clinic or something like that. Somebody was watching you do the exercises.” (C7)
Mode of delivery	Regular contact via telephone calls	“I think that worked really well because the video calls were kind of a bit of a hassle to set up and that kind of thing, whereas the phone calls made it really accessible, but the videos gave you greater information and allowed you to do more with your clients.” (S7) “I’ve actually found it pretty useful, mainly because the phone call every week tends to keep me honest about what I’m doing, and I don’t want to tell too many fibs about it.” (C3) “Having that phone call each week was really motivating. Because you’d get halfway through the week and go oops, I haven’t been doing what I’m supposed to do. Oops, they’re going to ask me, so you sort of had that guilt.” (C5) “So that’s why I think I stopped the phone calls, because I thought, it’s getting me nowhere and I’ll wait until the Hub (i.e., classes) starts up again. But then I thought to myself, I’m not doing the exercises, and that’s why, I thought I’m wasting their time and mine.” (C6) “I wasn’t looking forward to those phone calls. ……… And sometimes they were half an hour later, right, so I’m hanging around, I can’t go outside, I can’t do this, I can’t do that.” (C1)
	Using video calls	“A bit daunting to begin with, because I hadn’t done it before, hadn’t set it up, logged in, and it just worried me, something would go wrong. Once I got in to do it, it was fairly straightforward.” (C8) “So, it shows that people, if they can … if we can teach them a system, they’re pretty good at learning that technology.” (S2) “The video compared to the phone was a lot more technologically challenging for us and the clients …… It’s a lot more time consuming, especially video calling.” (S4)
Therapeutic relationships	Maintaining relationships	“So we sort of interspersed it with a little bit of conversation and I think that’s really important that the students don’t mind doing that. I know it’s a phone call and it’s a very specific phone call they’re making, but all those little bits actually help to build that relationship.” (C5) “The students were all very good, very friendly and genuinely interested in what you were doing.” (C7)
	Challenges of building relationships via telephone calls	“…. you had the phone call and you’re just answering the questions that they ask you, you can’t see the person, so you miss the interaction that you have.” (C10) “So some people would be like, “oh, [student name] you’re back again.” And then other people would just have no idea that I was the same person that had called them like beforehand.” (S6)
		“And with changing the students, that was difficult. So when we first started off it was [student name] on the phone, and because we’d worked with [student name] I knew the person. Right. It was very difficult to get a new student, and sometimes the student wasn’t exactly sure just how to approach over the phone.” (C1)

#### Empowerment

Clients who successfully engaged in telehealth (i.e. adhered to the prescribed exercise, participated in telehealth appointments, experienced no adverse events) displayed some common qualities, including a feeling of agency over their exercise program. They had ownership of their exercise program, including choosing their preferred mode of delivery, which was possible due to the flexibility of the service. Those clients who were empowered were able to solve problems like a lack of equipment at home.

“Just found a suitable location to bounce the ball inside of the house or I got an old chair and made that up to sit on, and move up and down, and I did the bungee straps on the bikes in the shed, and pulled them back and forth, just improvising all the time.” (Client (C) C2)

They demonstrated more flexibility in their performance of the home exercise program, developing strategies to self-monitor their exercise, including recording sheets to track progress, made feedback inherent in the exercise and utilised technology, reducing the need for specific feedback from the therapist.

#### Feedback

The theme of feedback encapsulated both the feedback provided on the exercise program and the high value clients placed on receiving feedback on their performance. Receiving feedback on exercise performance was consistently reported as one of the main benefits of in-person classes.

“So that’s what I like best of all, you’ve got somebody telling you what to do, and they watch what you do.” (C11)

Clients and students noted that while telephone calls allowed clients to be connected and provided feedback on adherence, they did not allow specific feedback on exercise performance. This led to some misunderstanding about how to perform the exercises.

Clients who took part in individual video calls reported they enabled greater feedback, enhancing their experience. Many felt that the service could be improved by providing additional individual video calls.

“They were asking me to do different things, and I felt like they could see what I was doing, and they could be accurate in their assessment of what was happening. ….. And they would be tweaking advice about doing it in a different way, or a better way. So again, I think it worked pretty well actually, considering it was all done on video.” (C9)

#### Mode of delivery

The ability to provide different modes of telehealth (telephone, individual and group video calls), combined with written and illustrated exercise programs was valued, as clients’ telehealth preferences varied. The group video calls were perceived as the least successful. This was due to difficulties with technology e.g. poor internet connection and the dual tasking required to watch the screen and exercise at the same time. Some clients found it too distracting to see themselves, the therapist, and the other clients on the screen simultaneously. Also, video calls were not suitable for all clients due to safety concerns.

“Sometimes it was the audio, for sound. And then sometimes I lost it for whatever reason. And then others came in and they disappeared because of the videos, they didn’t always have good reception, so it was waiting while somebody else went to another room to try to come back.” (C1)

Both clients and students reported that telephone calls were more accessible, efficient and simpler than video calls and *“kept clients motivated and in touch.”* However, for some weekly telephone calls were a hassle, led to frustration and were not perceived as helpful, especially if they were not completing the exercises. Telephone calls also had limitations when students were prescribing exercises for clients they had not seen.

“So that’s why I think I stopped the phone calls, because I thought, it’s getting me nowhere and I’ll wait until the Hub [in person group exercise class] starts up again. But then I thought to myself, I’m not doing the exercises, and that’s why, I thought I’m wasting their time and mine.” (C6)

The individual video calls overcame these limitations, provided the students with more information than telephone alone but required more time, and some clients reported initially feeling *“daunted and worried”* by the technology. Once over the initial challenges, clients reported that individual video calls were effective as they could complete the exercise and then be seated to receive feedback, so dual tasking was not required.

Clients felt that attending the clinic prior to telehealth was beneficial as it gave them confidence to exercise at home: *“some of the exercises I knew because we had done them in class.” (C5).* They understood the importance of exercise and how to complete their program. Many felt a combination of in-person and telehealth would be optimal.

“If you only have the home exercise classes without having built up some sort of routine or a set of expectations around which you’re going to do the exercises, I think it would be less successful. I think a combination of the two would be the better of the two. And just going straight into home exercises without having built up that initial framework of exercises and a habit and a motivation, I think it would be less successful.” (C13)

#### Therapeutic relationship

Therapeutic relationship encompassed the relationships between clients and the therapist and/or students. For some clients, these relationships were well established from clinic attendance. However, relationships changed during telehealth as the students changed and, for new clients, relationships had to be developed over telehealth. The therapeutic relationship influenced clients’ experiences and engagement with telehealth. For some clients, regular contact with the telephone calls was an effective way to stay connected and maintain relationships.

“If I hadn’t any sort of interaction with the people [physiotherapist and physiotherapy students] there, I probably would have done things like wouldn’t have done the exercises, I don’t think.” (C10)

Students reported that the ease of building therapeutic relationships using telehealth varied from client to client. Interactions between the clients, therapist and/or students via telephone were shorter than in person and there was no visual information to assist communication. The non-motor impairments associated with Parkinson disease may also have contributed to difficulties in some cases.

### Integration of file audit and interview results

The positive experience of telehealth for most clients may have contributed to the high adherence to the home-exercise program and maintenance of physical activity. As most of the clients had attended the clinic prior to telehealth they had developed therapeutic relationships, had greater understanding of the exercises and received feedback on performance prior to exercising at home. These factors may have improved the clients’ sense of empowerment and contributed to the high adherence and maintenance of physical activity observed in this study. The regular contact via telehealth also aided adherence as clients were accountable to the therapist each week. However, telehealth was not suitable for all clients. Despite multiple modes of delivery some clients found it difficult to engage with telehealth. Those clients who did not engage in telehealth were more likely to have low adherence or stop completing the home-exercise program.

## Discussion

Physiotherapy delivered via telehealth as part of clinical practice enabled clients to perform their home exercise program and maintain physical activity despite having no access to exercise classes during COVID-19 restrictions. Telehealth, utilizing a combination of weekly telephone calls and individual or group video calls, was key to achieving this with minimal adverse events. Telehealth relied on a flexible approach from both clients and staff. When developing and delivering telehealth, the important components to consider were how to: empower clients, provide feedback, build and maintain therapeutic relationships and determine the optimal individual mode(s) of telehealth delivery.

Overall, the average amount of time completing the prescribed exercise (101 min per week) was less than the World Health Organisation guidelines for physical activity for older adults of 150 min per week of moderate-intensity physical activity [1]. However, it seems likely that many clients did meet these guidelines as the average step count per day was relatively high, at 11,305 steps. Especially when compared to the average daily step count reported for PwP (5,876 per day) during the first wave of COVID-19 in Sweden [9].This suggests that many clients were being physically active beyond the home-exercise program.

This study utilized three different types of telehealth; telephone, individual video and group video calls. Telephone calls were used because they did not require any additional technology, were familiar to clients and had been used previously to support home-based exercise [2, 20]. Individual video calls were important to teach new clients their home-exercise program. Individual video calls also provided clients with feedback on their exercise performance and assisted in developing therapeutic relationships. This is consistent with previous studies which reported video calls were an effective way to monitor home-based exercise [21, 22].

The group video calls aimed to replicate the social interaction which occurred when clients were exercising in-person in classes [23]. However, we found group calls were impractical due to technology issues and difficulty individualising exercises. Clients had to dual task; exercising and watching the screen at the same time, which is challenging for PwP and may have contributed to one client falling. Previous telehealth studies have minimised this risk by incorporating seated exercise [2, 22] or having another person present for safety during exercises [21]. There was also minimal interaction between clients during the call, possibly because clients had no additional capacity to engage with others while dual-tasking, lacked confidence with the technology or familiarity with communicating in a virtual environment.

Physiotherapy provided via telehealth was safe for people with mild to moderate Parkinson disease. There were only two adverse events during home exercising. This is comparable to previous in-person research with similar clients conducted by our team where two falls occurred during a 10-week period [20]. It is important to note that physiotherapy via telehealth may not be suitable for people with severe Parkinson disease. In this study, those with more severe disease, especially those with cognitive impairment, were not offered telehealth, as it was deemed unsafe for them to exercise without a trained supervisor. Previous research has shown that minimally supervised home-based exercise can increase falls in people with more severe Parkinson disease [24–26]. Selection of clients for telehealth needs to be carefully considered, taking into account the amount of additional supervision by partners/carers which may be required.

This study has provided some key learnings regarding the requirements for the successful implementation of physiotherapy provided via telehealth. Firstly, the selection of both suitable people and the appropriate mode of telehealth is vital. Most people who have mild to moderate Parkinson disease are able to safely and effectively participate in telehealth. Having multiple modes of telehealth allows the therapist and PwP to take into consideration their access and ability to use technology. Where possible, the use of individual video calls is recommended, as the therapist can gather information from observing the person performing the exercise, provide feedback on the performance of exercise and build therapeutic relationship. It is also noted that in some jurisdictions, video calls may be required in order to meet the requirements of a consultation. Physiotherapists can use telehealth to empower people, giving them agency and ownership of their exercise and creating shared decision making. Finally, telehealth could be combined with in-person physiotherapy. The advantage of this hybrid model of care is that therapeutic relationships can be developed, PwP have a greater understanding of the exercise and there are opportunities for feedback on the performance of the exercises prior to exercising at home.

This study has some limitations including the use of retrospective data for the file audit. While every attempt was made to gather the physical activity data as soon as COVID-19 restrictions occurred some clients had undertaken telehealth for some weeks prior to physical activity being measured. Further, this study occurred in a city where in-person services were only restricted for 21 weeks with minimal COVID-19 in the community. These factors combined with the small number of interviews conducted may limit the generalisability of the data. The use of mixed methods is important as the quantitative data was collected retrospectively and adherence was self-reported. The qualitative interviews increased depth of the data and provided a comprehensive insight into clients’ and students’ experiences.

## Conclusion

This study shows that telehealth can be used effectively in the clinical setting to support PwP to exercise and maintain their physical activity. Group exercise provided via telehealth needs further exploration in PwP to ensure safety. Furthermore, the use of telehealth may be optimised when combined with in-person physiotherapy in a hybrid model of care as occurred with most clients attending the centre prior to undertaking telehealth. While there is emerging evidence that a hybrid model of care is feasible and acceptable [20, 27], further research is required to explore the efficacy of this model. A hybrid model of care has the potential to transform current physiotherapy management of Parkinson disease beyond the COVID-19 pandemic by providing long-term, sustainable and accessible physiotherapy.

## Supplementary Information

**Additional file 1:** **Supplementary material 1.** Example of home exercise program.

**Addtitional file 2:** **Supplementary material 2.** Explanation of the telehealth service provided.

**Additional file 3:** **Supplementary material 3.** Semi structured interview questions.

## Data Availability

The analyzed datasets are available from the corresponding author upon reasonable request.

## Abbreviations

COVID-19: Corona Virus Disease of 2019
PwP: People with Parkinson disease
IPAQ-E: International Physical Activity Questionnaire – Elderly
