# Supplementary material for: Utilising telehealth to support exercise and physical activity in people with Parkinson disease: a program evaluation using mixed methods
Source: BMC Health Serv Res. 2023 Mar 7;23:224. doi: 10.1186/s12913-023-09194-0 (PMC9991450; doi:10.1186/s12913-023-09194-0)
Supplement: Supplementary file 1 — Additional file 1: Supplementary material 1. Example of home exercise program. [file 12913_2023_9194_MOESM1_ESM.pdf]

Supplementary material 1: Example of home exercise program

Additional file 1

- File format: Portable Document Format .pdf
- Title: Example of home exercise program including recording sheet
- Description: Example of a home exercise program and recording sheet given to a client during the telehealth service.

1. Big step using visual cues – Warm up

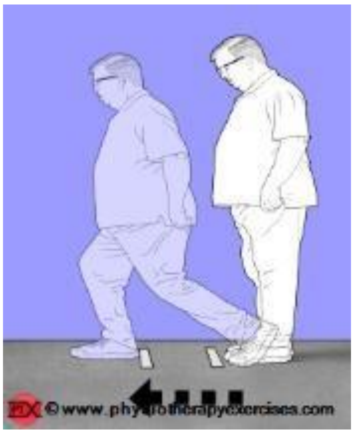

Client`s aim

To improve your ability to take big steps.

Client`s instructions

Position yourself in standing with two markers indicating the size of the step required (40 cm apart). Step forward so that you step over both markers.

Repeat 5 times on each leg

2. Bilateral calf raises – Warm up

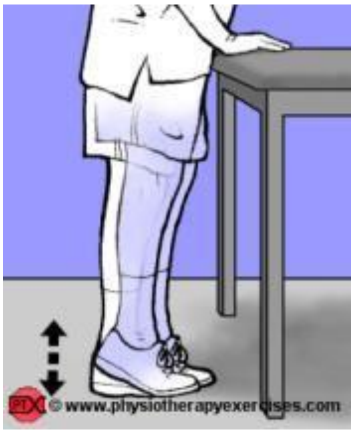

Client`s aim

To strengthen your calf muscles.

Client`s instructions

Position yourself standing with your feet together. Start with your heels on the ground. Finish with your heels off the ground.

2 x 15

3. Stand up and sit down – Warm up

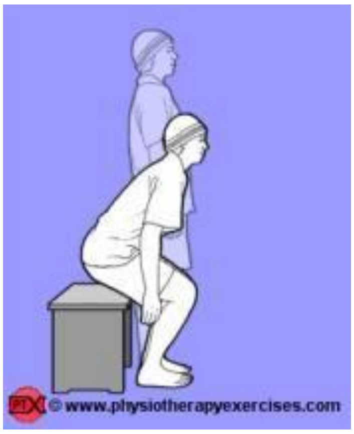

Client`s aim
To improve your ability to balance while standing up and sitting down
.

Client`s instructions
Position yourself sitting with your toes under your knees. Practice standing up and sitting down. Ensure that your shoulders and knees move forward quickly to stand up and stick your bottom out to sit down. Ensure you stand all the way up and sit all the way down.

2 x 15

4. Step on spot with high knees using visual cues – Warm up

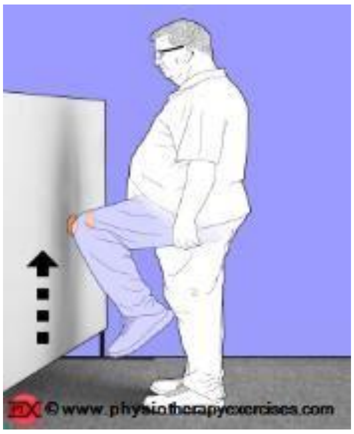

Client`s aim
To improve your ability to lift the knees up and balance.

Client`s instructions
Position yourself in standing with targets at hip height, on a wall in front of you. March on the spot touching your knees to the targets. Ensure you stand up tall.

Perform for 30 seconds

5. Reach in multiple directions

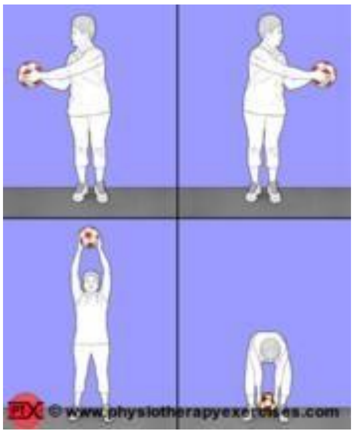

Client`s aim
To improve your ability to turn and reach in multiple directions.

Client`s instructions
Position yourself in front of a wall, standing holding a ball. Turn from side to side, stretching so that the ball touches the wall. Then, reach up to touch the wall above your head and squat down to touch wall between your feet.

Repeat 10 times in each direction

Do NOT perform reaching to the ground

6. Walk with big steps and big arms

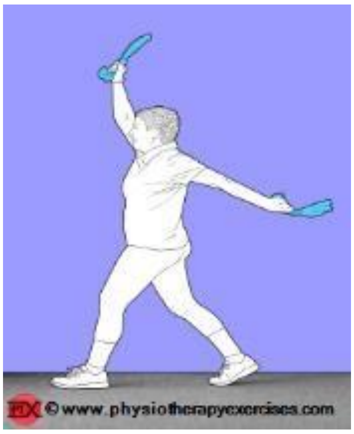

Client`s aim
To improve your ability to walk with big steps and big arm movements simultaneously
.
Client`s instructions
Hold a piece of ribbon in each hand. Walk forward with big steps, swinging the arms as high as possible with each step, i.e., exaggerate your arm swing while walking.

Perform for 5 – 10 minutes

7. Step up

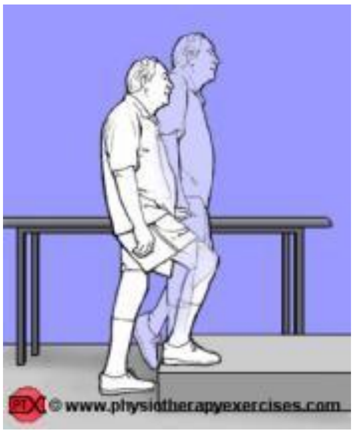

Client`s aim
To improve your ability to step up and balance.
Client`s instructions
Position yourself in standing with a block in front of you, both feet on the ground and a stable support nearby. Practice stepping up on to and off the block, one foot at a time. Ensure that hand support is used only if you feel unsteady.

3 x 10 on each leg

8. Step and turn on the spot

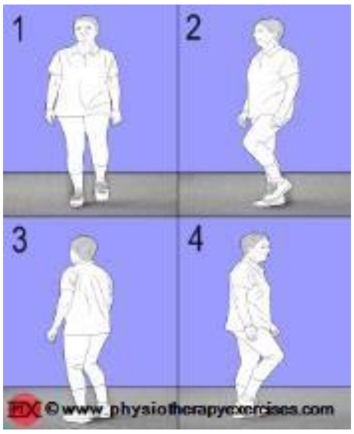

Client`s aim
To improve your ability to turn on the spot
.
Client`s instructions
Position yourself in standing and begin stepping on the spot. Then, keep stepping while turning 180 degrees. Repeat in the opposite direction. Aim for 4 steps for a full 180 degree turn. Ensure your steps are high enough to clear the ground.

Repeat 5 times in each direction

Make sure you perform this next to something stable

9. Step over an obstacle

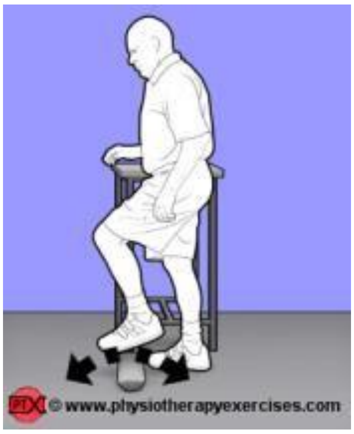

Client`s aim
To improve your ability to step over obstacles and balance.

Client`s instructions
Position yourself standing with an obstacle on the floor in front of you and stable support nearby. Practice taking a big step over the obstacle and shift your weight onto your forward leg, before stepping back. Ensure that hand support is used only if you feel unsteady.

2 x 10 on each leg

10. Step sideways over an obstacle

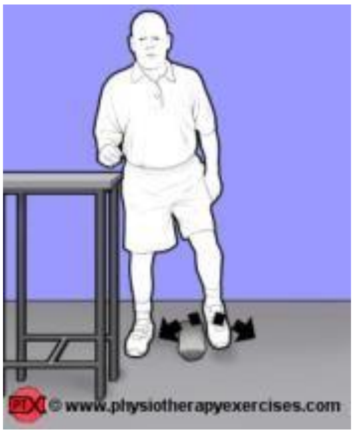

Client`s aim
To improve your ability to step over obstacles and balance.
Client`s instructions
Position yourself standing with an obstacle on the floor beside you and stable support nearby. Practice taking a big step sideways over the obstacle and transfer your weight onto the stepping leg before stepping back. Ensure that hand support is used only if you feel unsteady.

2 x 10 on each leg

Physical activity record sheet

Please record time in minutes and type of other exercise

Week Starting	Activity	Monday (minutes)	Tuesday (minutes)	Wednesday (minutes)	Thursday (minutes)	Friday (minutes)	Saturday (minutes)	Sunday (minutes)
**Eg.**	Exercise program	20		30		20		
**13/04/20**	Other exercise		Walking - 90		Yoga 20		Mowing the lawn - 30	
	Exercise program							
	Other exercise							
	Exercise program							
	Other exercise							
	Exercise program							
	Other exercise							

- Other exercise could include gardening, walking, cycling for example
- Need to be in bouts of 10 minutes or more
