# Supplementary material for: Utilising telehealth to support exercise and physical activity in people with Parkinson disease: a program evaluation using mixed methods
Source: BMC Health Serv Res. 2023 Mar 7;23:224. doi: 10.1186/s12913-023-09194-0 (PMC9991450; doi:10.1186/s12913-023-09194-0)
Supplement: Supplementary file 2 — Addtitional file 2: Supplementary material 2. Explanation of the telehealth service provided. [file 12913_2023_9194_MOESM2_ESM.pdf]

Additional file 2

- File format: Portable Document Format .pdf
- Title: Detailed explanation of telehealth service provided.
- Description: Further information about the telehealth service which was provided

Supplementary material 2: Explanation of the telehealth service provided

During the first five weeks the region was in a lockdown where only essential services were available, and people aged over 65 were encouraged to stay at home. There were no in-person exercise classes for the first 14 weeks. In the final seven weeks, in-person exercise classes gradually resumed but with fewer clients.

Transition of clients to telehealth

People with Parkinson disease who were attending the physiotherapy group exercise classes were transitioned to a home-based exercise program supported by physiotherapy via telehealth. The transition involved the following:

1. Telephone screening to determine each client's willingness and suitability to be involved in telehealth.
    - Those clients with severe Parkinson disease, including significant cognitive impairments, who were attending the clinic prior to the telehealth were not offered the telehealth service as it was deemed unsafe for them to complete mobility and balance exercise at home without a therapist present and there was no time to train a carer or partner.
2. The clients completed questionnaires about their current physical activity, (International Physical Activity Questionnaire – Elderly (IPAQ – E)).
3. Development of an individualised home exercise program focussing on mobility and balance by a physiotherapy student, with all programs reviewed by the physiotherapist

prior to being emailed or mailed to the client. Consideration was given to clients to goals, ability, and available equipment and exercises were selected for the home program from the PhysioTherapy eXercises website. This website has over 100 specific exercises for PwP and includes written instructions, illustrations and recording sheets.

- The home exercise program was updated by the physiotherapy students every three to five weeks based on feedback from the clients. All programs were reviewed by the physiotherapist prior to being email or mailed to the client.

4. Clients were offered the opportunity to wear a physical activity monitor (ActiGraph GT3X+) for one week during the initial weeks of telehealth service and another one week prior to returning to in-person exercise classes.
5. Clients completed the three questionnaires IPAQ-E prior to returning in person to the exercise classes.

Telehealth provided by telephone calls

Clients were contacted weekly via a telephone call which lasted for approximately 10-15 minutes per call. The telephone calls were conducted by the physiotherapist or physiotherapy students under the supervision of a physiotherapist. The calls included a discussion of the home-based exercise program and the client reported if they had been completing the exercise program and any challenges or barriers they had experienced. If barriers to completing the exercise were identified, the client and therapist develop solutions to overcome these such as identifying alternative equipment which could be used. The physiotherapist and/or students provided feedback, highlighted successes, provided encouragement, monitored, and progressed the exercise program as indicated. When indicated, exercises were progressed by increasing the dose, making the exercise more

challenging such as adding weight or reducing the base of support or changing the exercise. Every three to five weeks clients were sent an updated exercise program which reflected the changes made during the weekly telephone calls.

Where possible telephone calls were conducted at a similar time each week which corresponded to when the client would have been attending the clinic in-person.

Telehealth provided by individual video calls

Individual video calls were conducted using the online software Coviu (Coviu Global Pty Ltd, Australia). The client could see both the physiotherapist and themselves on their screen.

Clients were selected for individual video calls if they were a new client to the clinic (clients who did not attend any in-person group-based exercise classes prior to the implementation of telehealth) or based on the clinical judgement of the physiotherapist; for example if the client experienced significant difficulties when exercising at home, or increased in freezing of gait. Clients needed to have a device with a camera, microphone and stable internet connection to participate in an individual video call.

There were two main types of individual video calls conducted.

- An initial assessment of the new client. This included a client interview and assessment of the clients current physical abilities, where possible. (60 to 90 minutes).
- Teaching a client their home exercise program or a strategy to manage freezing of gait. This involved the physiotherapist or physiotherapy students under the

supervision of the physiotherapist demonstrating the exercise, including safety features (e.g., a stable surface nearby for support if required) while the client was sitting down watching the screen. The client then completed the exercise. Once the client had finished the exercise and was seated, the physiotherapist or physiotherapy student provided feedback on their performance. (30 to 60 minutes)

Telehealth provided by group video calls

Group video calls were conducted using the online software Coviu (Coviu Global Pty Ltd, Australia). This involved 3-4 clients on the same video call as the physiotherapist. Clients could see all the other clients and the physiotherapist on their screen. The class was led by the physiotherapist or physiotherapy student under the supervision of the physiotherapist.

The class involved a seated warm up followed by balance and lower limb strengthening exercises. The physiotherapist demonstrated each exercise while all the clients watched their screen, while seated. All the clients then completed the exercise at the same time with the physiotherapist and/or physiotherapy student watching the screen and providing feedback as the client was completing the exercise. There was a short 1–2-minute rest period between each exercise. This process was then repeated for each exercise.

Video group class lasted 60 minutes with approximately 40-45 minutes of exercise completed.

Clients were selected for the group video call based on the clinical judgement of the physiotherapist taking into consideration the client’s physical ability, cognition, willingness to be in a group environment, ability to use technology, and the clients request to be involved

in a group call. Clients needed to have a device with a camera, microphone and stable internet connection to participate in the group video call.

There were 15 group video calls conducted with 12 clients during the 21 weeks of telehealth.
