# Supplementary material for: Utilising telehealth to support exercise and physical activity in people with Parkinson disease: a program evaluation using mixed methods
Source: BMC Health Serv Res. 2023 Mar 7;23:224. doi: 10.1186/s12913-023-09194-0 (PMC9991450; doi:10.1186/s12913-023-09194-0)
Supplement: Supplementary file 3 — Additional file 3: Supplementary material 3. Semi structured interview questions. [file 12913_2023_9194_MOESM3_ESM.pdf]

Additional file 3

- File format: Portable Document Format .pdf
- Title: Box 1: Interview guide for qualitative interviews of clients and physiotherapy students.
- Description: A list of questions used for the semi-structured interviews with the clients and physiotherapy students to gather information about their experiences of telehealth.

Supplementary material 3 – Semi structured interview questions

Interview questions - Clients

1. Due to COVID-19 the UC Parkinson's clinic had to stop the exercise classes at the clinic. You were provided with a home-exercise program and weekly telephone or video calls during COVID-19. Tell me what was that like?
2. Can you tell me about the support you received while you were unable to attend the clinic? Can you think of other ways we could have improved support during this time?
3. Thinking about your experience of the home exercises and telephone/video calls, what were the good things? What were the most useful aspects? What did you **most** enjoy and why?
4. Thinking about your experience of the home exercises and telephone/video calls, what were the not so good things? What did you **least** enjoy and why?
5. Tell me about the technology that you used?
6. Tell me about your experience of completing the assessment via video call?
7. Overall, how satisfied were you with the home-based exercise and telephone/video calls and why?
8. Would you recommend the home-based exercise and phone calls to others with Parkinson's and why?
9. What advice would you give someone else with Parkinson's when completing home exercises and receiving telephone/video calls?
10. Tell me about your experience using the home exercise program instructions?
11. Tell me about your experience of recording the exercise your performed?
12. Is there anything else that you would like to add?

Interview questions – Physiotherapy students.

1. Due to COVID-19 the UC Parkinson's clinic had to stop the exercise classes at the clinic and rapidly transitioned to telehealth. Tell me what was that like?
2. Thinking about your experience of providing telehealth, what were the good things? What were the most useful aspects? What did you **most** enjoy and why?
3. Thinking about your experience of providing what were the not so good things? What did you **least** enjoy and why?
4. Tell me about the technology that you used?
5. What did you learn from the telehealth experience?
6. Overall, how satisfied were you with the providing telehealth and why?
7. Would you recommend telehealth to other Physiotherapist/ Physiotherapy students treating people with Parkinson's disease and why?
8. What advice would you give to someone else who was setting up a PD Telehealth clinic
9. Is there anything else that you would like to add?
